# Biodosimetry of Low Dose Ionizing Radiation Using DNA Repair Foci in Human Lymphocytes

**DOI:** 10.3390/genes11010058

**Published:** 2020-01-04

**Authors:** Lukáš Jakl, Eva Marková, Lucia Koláriková, Igor Belyaev

**Affiliations:** Cancer Research Institute, Biomedical Research Center, University Science Park for Biomedicine, Slovak Academy of Sciences, Dúbravska cesta 9, 845 05 Bratislava, Slovakia; eva.markova@savba.sk (E.M.); lucia.j.kolarikova@gmail.com (L.K.); igor.beliaev@savba.sk (I.B.)

**Keywords:** ionizing radiation, DNA double strand break, DNA repair foci, DNaseI

## Abstract

Purpose: Ionizing radiation induced foci (IRIF) known also as DNA repair foci represent most sensitive endpoint for assessing DNA double strand breaks (DSB). IRIF are usually visualized and enumerated with the aid of fluorescence microscopy using antibodies to γH2AX and 53BP1. This study analyzed effect of low dose ionizing radiation on residual IRIF in human lymphocytes to the aim of potential biodosimetry and possible extrapolation of high-dose γH2AX/53BP1 effects to low doses and compared kinetics of DSB and IRIF. We also analyzed whether DNaseI, which is used for reducing of clumps, affects the IRIF level. Materials and Methods: The cryopreserved human lymphocytes from umbilical cord blood (UCB) were thawed with/without DNaseI, γ-irradiated at doses of 0, 5, 10, and 50 cGy and γH2AX/53BP1 foci were analyzed 30 min, 2 h, and 22 h post-irradiation using appropriate antibodies. We also analyzed kinetics of DSB using PFGE. Results: No significant difference was observed between data obtained by γH2AX foci evaluation in cells that were irradiated by low doses and data obtained by extrapolation from higher doses. Residual 53BP1 foci induced by low doses significantly outreached the data extrapolated from irradiation by higher doses. 53BP1 foci induced by low dose-radiation remain longer at DSB loci than foci induced by higher doses. There was no significant effect of DNaseI on DNA repair foci. Conclusions: Primary γH2AX, 53BP1 foci and their co-localization represent valuable markers for biodosimetry of low doses, but their usefulness is limited by short time window. Residual γH2AX and 53BP1 foci are more useful markers for biodosimetry in vitro. Effects of low doses can be extrapolated from high dose using γH2AX residual foci while γH2AX/53BP1 foci are valuable markers for evaluation of initial DSB induced by ionizing radiation. Residual IRIF induced by low doses persist longer time than those induced by higher doses.

## 1. Introduction

DNA double-strand breaks (DSB) are the most detrimental DNA damages, which are cytologically manifested by DNA repair foci named also ionizing radiation-induced foci (IRIF) if induced by ionizing radiation [1]. The DNA repair foci are visualized as discrete fluorescent signals around the DSB by immunodetection of proteins associated with the DSB repair. One of the main events in the DSB repair is phosphorylation of H2AX, the variant of histone family 2A, that serves as a chromatin scaffold within a 2-Mb DNA domain recruiting the proteins involved in DSB repair [2]. The γH2AX protein has become a well-established biomarker for IRIF in low-dose research [3], diagnostic radiology [4,5,6], cancer research and therapy [7,8], and biological dosimetry [9,10,11]. Along with discrete foci, immunostaining with antibodies against γH2AX reveals so-called γH2AX pan-staining, which represents a visual manifestation of cells in early apoptosis [12]. As far as very few data are available on the γH2AX pan-staining induced by low-dose IR human lymphocytes in, we aimed to study it in this study.

The next useful molecular marker for DSB is a p53 binding protein 53BP1 [13,14], which is well-known promoter of the DSB repair by non-homologous end joining (NHEJ) [15,16,17]. While the γH2AX and 53BP1 are commonly accepted molecular markers for biological dosimetry and enumeration of DSB, they do not always co-localize with DSB due to different kinetics of γH2AX dephosphorylation and relocalization of 53BP1 from the location of the DSB [18,19]. Although co-localization of γH2AX/53BP1 is currently considered as the most reliable marker of the DSB, more research is needed to define limitations of γH2AX and 53BP1 assays for the DSB quantification [6]. In this study, we provide further evidence that both γH2AX and 53BP1 foci persist in cells a longer time than needed for the DSB repair.

Exposure of general population with low-dose ionizing radiation has tremendously grown in recent years. In particular, these exposures occur at airport security checks and in medical examinations such as mammography, computed tomography, dental X-ray exposures and angiography [5,20,21,22,23,24]. Biological dosimetry of low doses represents an important issue for radiation protection and prediction of possible health effects including cancer risks [25]. While most of the biodosimetric studies indicated linear dose response for IRIF, few of them provided evidence for increased sensitivity to low doses [18]. Remarkably, γH2AX and 53BP1 foci induced by low-dose IR in human fibroblasts and mouse tissues were reported to persist longer time than those induced by higher doses [26]. Human lymphocytes represent one of the most common cell types for biodosimetry. Thus, in this study we: (i) tested whether low-dose IR induced γH2AX/53BP1 foci in human lymphocytes could be extrapolated from the effects obtained with the high doses; (ii) compared the time kinetics of γH2AX and 53BP1 after low and higher doses to validate whether low and high dose IR induced IRIF persist for different post-irradiation time.

DNaseI is systematically used for preventing the cell clumps formation in fluid cell suspensions, which are analyzed by flow cytometry, imaging flow cytometry, etc. Application of DNaseI is particularly helpful for increasing the output of cryopreserved human lymphocytes after thawing. Free DNA from damaged cells with perforated membranes binds to intact cell membranes and causes the clumps formation. Ganassi et al. (1989) treated Chinese hamster fibroblasts with DNase I, which resulted in cell death and induction of chromosome aberrations at all stages of the cell cycle [27]. A known pathway how the DNaseI can damage genome is through the action of linear alkylbenzene sulfonates (LAS), a major type of anionic surfactant, which have been regarded as not genotoxic and exist at low concentrations in the environment. LAS disrupt the cytoskeleton releasing DNase I from filamentous actin. The released DNase I is translocated to the nucleus and breaks DNA [28]. To the best of our knowledge, there is no information about the effect of DNaseI on the level of DNA damage in human lymphocytes. In this study, we tested whether the use of DNaseI for cell thawing could have an effect on endogenous and radiation-induced damage in lymphocytes from umbilical cord blood.

## 2. Materials and Methods

Reagent grade chemicals were obtained from Sigma-Aldrich (St. Louis, MO, USA) and Merck KgaA (Darmstadt, Germany). PFGE-grade agarose, *Schizosaccharomyces pombe* and *Saccharomyces cerevisiae* markers were from BioRad (Richmond, CA, USA). Proteinase K was purchased from Boehringer Mannheim (Scandinavia AB, Bromma, Sweden).

This study has been approved by the Ethics Committee of Children’s Hospital in Bratislava (ICH GCP 135/95). Mononuclear cells (MNC) were extracted from umbilical cord blood (UCB) of 3 healthy newborns (2 boys and 1 girl) after full-term pregnancies as previously described [29], all samples were processed since 2 h up to 19 h after birth.

Frozen in liquid nitrogen UCB MNC (1 mL) were thawed and diluted in either (A) 4 mL of basal medium (BM): Roswell Park Memorial Institute medium (RPMI) 1640 medium with L-glutamine and 4-(2-hydroxyethyl)-1-piperazineethanesulfonic acid (Hepes) (PAA Laboratories GmbH, Pasching, Austria) supplemented with 10% fetal bovine serum (FBS), 100 IU/mL penicillin, 100 μg/mL streptomycin (Gibco, Invitrogen, Germany) or (B) 3.5 mL RPMI and 0.5 mL Hanks’ Balanced Salt Solution (HBSS) (Gibco, Invitrogen, Karlsruhe, Germany) containing 1 mg/mL DNaseI. Adherent cells were excluded after a 2 h incubation of MNC. Viability of remaining lymphocytes was not less than 95% as defined by the Trypan blue exclusion assay.

UCB lymphocytes were irradiated with 5, 10, and 50 cGy (and also with non-irradiated control) of ^60^Co γ-rays (dose rate from 0.461 to 0.501 Gy/min) on ice using the Theratron Elite 100 source (Mds Nordion, Ottawa, ON, Canada). Control cells were sham-irradiated. After irradiation, the cells were briefly pre-warmed at 37 °C and maintained 30 min, 2 h, and 22 h in BM at a humidified incubator at 5% CO_2_ and 37 °C (Sheldon Manufacturing Inc., Cornelius, OR, USA).

Upon having been incubated for preset time, the cells were washed with phosphate-buffered saline (PBS, 3.2 mM Na2HPO4, 0.5 mM KH2PO4, 1.3 mM KCl, 135 mM NaCl, pH 7.4) and spun down 5 min on the double cytoslides coated with polysine (Thermo Fisher Scientific Inc., Shandon, CA, USA) at 85 g using Cytospin 2 cytocentrifuge (Shandon). Then the cells were fixed with 3% paraformaldehyde in PBS for 15 min, permeabilized with 0.2% Triton X-100 in PBS for 5 min, washed extensively with PBS and blocked in 5% BSA in PBS (Gibco, Invitrogen, Karlsruhe, Germany) for 30 min at room temperature (RT). The primary antibodies, monoclonal mouse γH2AX (cat.# NB100-78356, Novus Biologicals, Abington, EN, United Kingdom) (dilution 1:400) and polyclonal rabbit 53BP1 (cat.# NB100-304, Novus Biologicals, Abington, EN, United Kingdom) (dilution 1:800), were diluted in 1% BSA in PBS and applied in 100 µL aliquots to the slides. The slides were incubated for 1 h in a humidified chamber at RT. After washing 3 times with PBST (PBS containing 0.1% TWEEN^®^ 20, Sigma-Aldrich (St. Louis, MO, USA), the secondary antibodies, Alexa Fluor 488 IgG (H+L) antimouse (cat.# A-10680, Invitrogen Molecular Probes, Life Technologies, Waltham, MA, USA) and Alexa Fluor 555 IgG (H+L) antirabbit (cat.# A27039, Invitrogen Molecular Probes, Life Technologies, Waltham, MA, USA) (both in dilution 1:200), were added. Then, the slides were incubated for 1 h in a humidified light-protected chamber at RT, washed 3 times with PBST, and counterstained with antifade reagent agent Vectashield (Vector Laboratories, Burlingame, CA, USA) containing 4′,6-diamidino-2-phenylindole (DAPI). All experiments were performed in three replicates.

**Figure 1:**
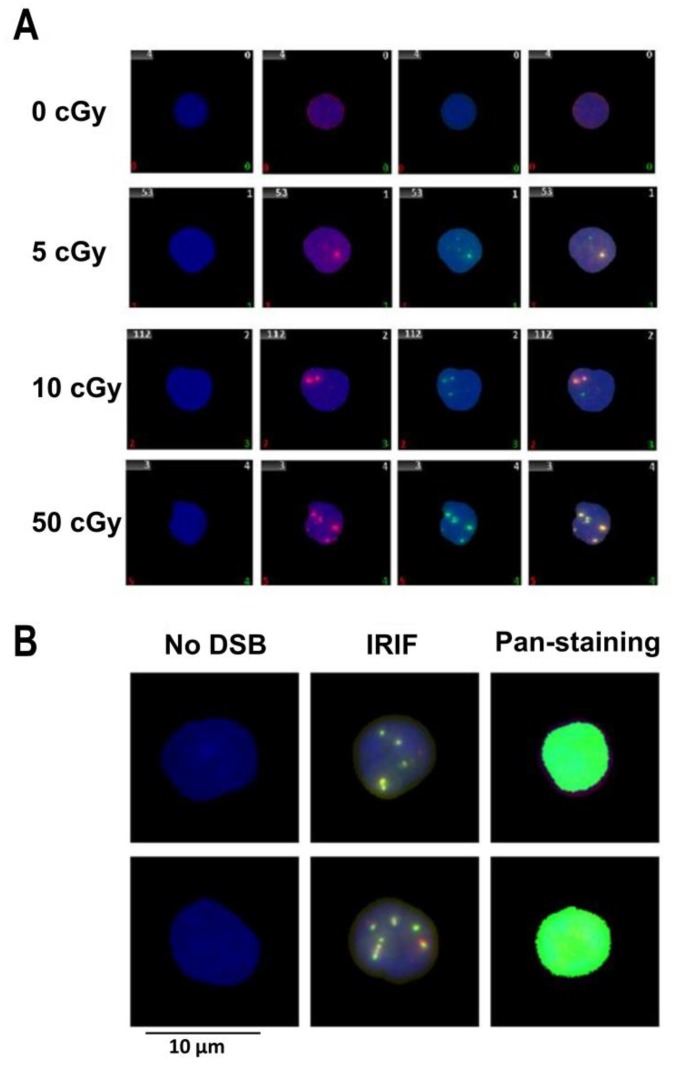
Representative images of nuclei from human UCB. Representative images of: (**A**) the nuclei (stained with DAPI in blue) from the human cord blood lymphocytes 30 min post-irradiation with 0 (sham irradiated control), 5, 10, and 50 cGy: γH2AX foci (green), 53BP1 (red), co-localized γH2AX/53BP1 overlay of green and red (yellow); and (**B**) representative raw images of pan-nuclear staining of γH2AX observed in human cord blood lymphocytes 22 h post-irradiation by the dose of 50 cGy.

Image acquisition of the DSB repair foci were conducted using the METAFER Slide Scanning System (MetaSystems, Altlussheim, Germany) based on Zeiss Axioscop 2 epifluorescent microscope (Carl Zeiss Microscopy, Jena, Germany), main parameters being: objective magnification (63x), number of focus planes (10), and focus plane distance (28/40 μm). Analysis of 53BP1, γH2AX, co-localized 53BP1/γH2AX foci (Figure 1A) and pan-nuclear γH2AX stained cells (Figure 1B) was performed with optional manual correction in individual cells as previously described [12]. At least 250 cells per slide and field in total at least 1000 cells were analyzed for each exposure condition.

After irradiation, the cells were washed with PBS, embedded into low melting point agarose, and lysed at 37 °C for 48 h in 0.5 M EDTA (pH 8.0), 1% sarcosyl, and 1mg/mL proteinase K as previously described [30]. Electrophoresis was carried out at 14 °C in 0.5× Tris-borate-EDTA (TBE) buffer with a CHEF-DR II apparatus (Bio-Rad) using the following steps: (1) 48 h at 35 V; (2) 48 h at 50 V; a 96 h pulse ramp overlapped these two steps with pulses decreasing from 90 min to 45 min; (3) 48 h at 60 V with pulse ramp from 45 min to 2 sec. After the electrophoresis, gels were stained with 0.5 µg/mL ethidium bromide and images were acquired at appropriate saturation using a CCD-camera (BioRad). The integrated optical density (IOD) of DNA released into the lanes was analyzed using QuantiScan for Windows software version 3.0 (Biosoft, Cambridge, UK).

The data were analyzed by the factorial analysis of variance (ANOVA) and the false discovery rate (FDR) using Statistica version 8.0 (Statsoft) and Microsoft Excel. Comparison between the groups was performed using a two-tailed t-test. The results were considered as significantly different at *p* < 0.05. The IRIF (radiation-induced excess overvalues of unirradiated control) observed after irradiation was approximated by linear regression Y = βD, which was validated by the coefficient of determination R^2^.

## 3. Results

In this study, 53BP1, γH2AX, co-localized 53BP1/γH2AX foci (Figure 1A) and γH2AX pan-staining (Figure 1B) were analyzed at 30 min, 2 h, and 22 h after irradiation with doses of 5, 10, 50 cGy and in sham-irradiated control cells (0 cGy). We analyzed cells with and without DNaseI treatment during thawing to verify whether this treatment, which significantly increases the output of cells after the freezing-thawing cycle, affects the level of the end-points studied.

### 3.1. Effects of Factors: Dose, Post-Irradiation Time, DNase

**Figure 2:**
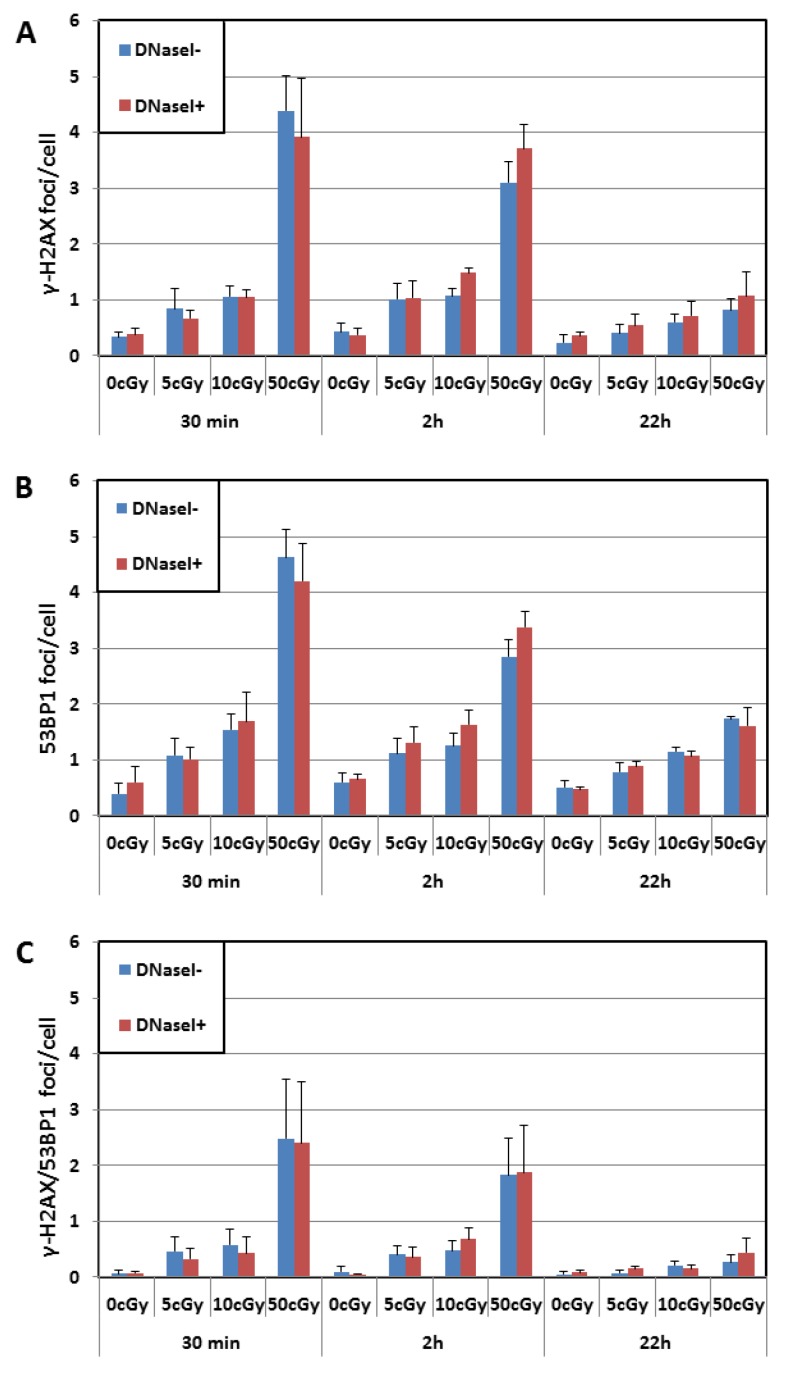
The level of γH2AX foci (A), 53BP1 foci (B), and co-localized γH2AX/53BP1 foci (C) in the cells thawed with and without DNaseI. Cells were thawed with or without DNase irradiated with doses 0, 5, 10, and 50 cGy and immunostained 30 min, 2 h, and 22 h post-irradiation. Each data point shows the mean value and standard deviation from 3 experiments.

**Figure 3:**
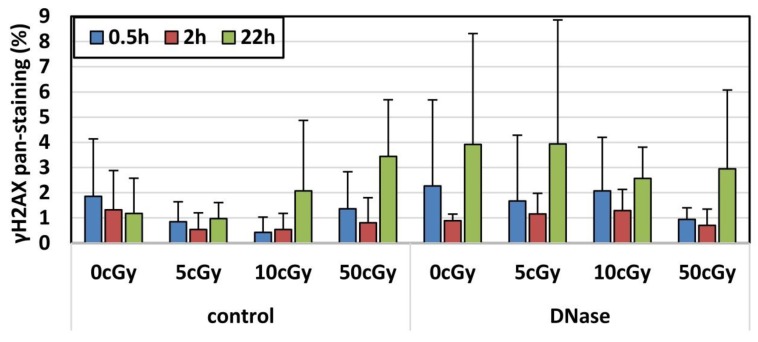
The level of γH2AX pan-staining in nuclei of cells 0.5 h, 2 h, and 22 h post-irradiation with doses 0, 5, 10, and 50 cGy. The figure shows the level of γH2AX pan-staining by the dose. The figure shows mean values and bars represent standard deviations.

The numbers of DNA repair foci obtained at different doses are shown in Figure 2A–C. We analyzed the obtained data using multifactorial multivariate ANOVA to reveal a possible dependence of output on a dose, time post-irradiation time, and DNaseI treatment (Table 1). We found that both dose and post-irradiation time statistically significantly affected the level of 53BP1, γH2AX, and co-localized 53BP1/γH2AX foci. On the contrary, γH2AX pan-staining did not depend on dose and time (Figure 3). Thus, our data indicate that the γH2AX pan-staining is not a valuable marker for biodosimetry.

No significant effect of DNaseI was found on the level of 53BP1, γH2AX, and co-localized 53BP1/γH2AX foci at any post-irradiation time (30 min, 2 h, and 22 h) regardless of irradiation dose. We did not observe the impact of DNase I on the γH2AX-pan-staining level either (Figure 3). After being proved that the treatment with DNase I did not affect the yield of IRIF/γH2AX pan-staining, we next analyzed radiation response and time kinetics using the merged DNase I+ and DNase I- data.

### 3.2. Dose Response for IRIF

Foci, which are visualized up to 4–5 h post-irradiation, are usually classified as primary foci. In contrast, residual foci can persist much longer time and usually analyzed at ≥20 h [31]. For assessment of the sensitivity of lymphocytes to low-dose radiation at the level of primary and residual IRIF, we performed statistical analyses comparing effect at each dose with sham-irradiated control (Table 2). Regardless of molecular marker (53BP1, γH2AX, or co-localized 53BP1/γH2AX), we observed a significant effect of all doses on primary IRIF at the time-points of 30 min and 2 h (Table 2). While 53BP1 residual foci still persisted 22 h post-irradiation with all doses, γH2AX and co-localized 53BP1/γH2AX residual foci were only detected at the doses of 10 and 50 cGy. In accordance with previous observations [30,31,32], this data suggested that the 53BP1 is a more sensitive marker for the evaluation of low-dose-induced residual DNA repair foci in human lymphocytes. In line with the aforementioned multifactorial analysis, we did not find any effect of dose on the level of γH2AX pan-staining.

**Figure 4:**
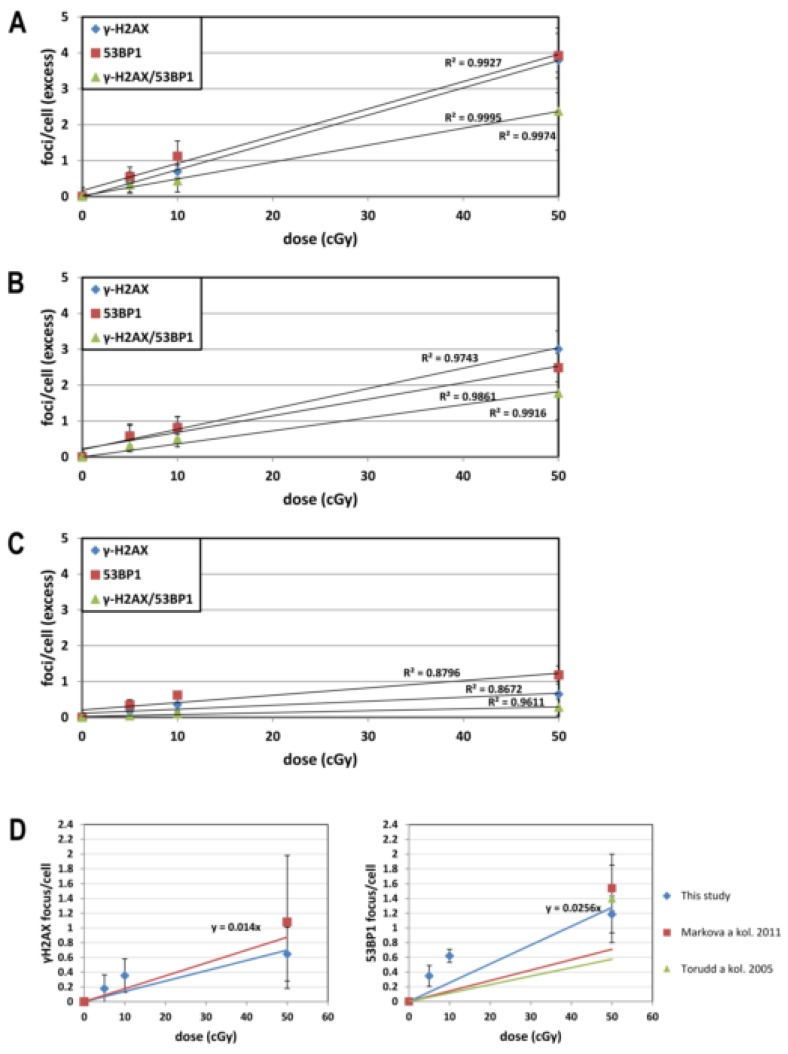
Linear fit for dose dependence of γH2AX, 53BP1, and γH2AX/53BP1 foci. Dose dependence of IRIF and linear regression along with the coefficient of determination R^2^ is shown for different post-irradiation time points: (**A**) 30 min, (**B**) 2 h, and (**C**) 22 h. Data from 3 experiments are shown for each dose (0, 5, 10, and 50 cGy). Panel (**D**) shows linear responses for residual foci measured 22 h post-irradiation (blue) and our extrapolations from high doses reported by Markova et al. [31] (red) and Torudd et al. [32] (green).

The obtained dose responses for radiation-induced 53BP1, γH2AX, and co-localized 53BP1/γH2AX foci fitted well to linear dependences as shown along with R^2^ in Figure 4A–C. The yield of foci per cGy per cell counted for each dose and derived from the slope Y = β D was calculated for each time point (Table 3) and shown along with coefficient of determination in Figure 4A–C.

The dose-response for residual IRIF induced in human lymphocytes by high doses ≥ 50 cGy has previously been studied for γH2AX [31] (1.75 ± 0:37 focus/cell/Gy) and 53BP1 [31,32] (1.42 ± 0:08 and 1.15 focus/cell/Gy). We found, that the results obtained here for residual IRIF at the dose 50 cGy are in line with data from the same dose in the studies of Markova et al. [31] and Torudd et al. [32] (Figure 4D). We further verified whether IRIF data obtained in this study with low doses fit those obtained by extrapolation of previously obtained results with higher doses. Dose dependence for γH2AX residual foci in cells irradiated by low-dose IR (Figure 4D) was also in line with extrapolated dose-dependence from high doses [31]. On the contrary, our 53BP1 data fitted by a linear response with a steeper slope as compared to the extrapolation from higher doses, which was obtained by recalculation from effects of the moderate dose of 50 cGy and the higher doses of 1 Gy and 2 Gy (Figure 4D). As Figure 4 shows, the IRIF levels induced by low doses ≤ 10 cGy outreach the trend line obtained for the dose range ≤ 50 cGy (Figure 4). The data suggested that the residual IRIF induced by doses ≤ 10 cGy persist longer in comparison with foci induced at higher doses. For this reason, the dose responses were calculated separately for doses ≤ 10 cGy and ≤50 cGy in Table 3 and time kinetics was further analyzed for IRIF at each dose. The linear dose response slope for each biomarker calculated for doses ≤ 10 cGy was significantly higher than the same linear response obtained by doses ≤ 50 cGy at 22 h (Table 3).

### 3.3. Time Kinetics

**Figure 5:**
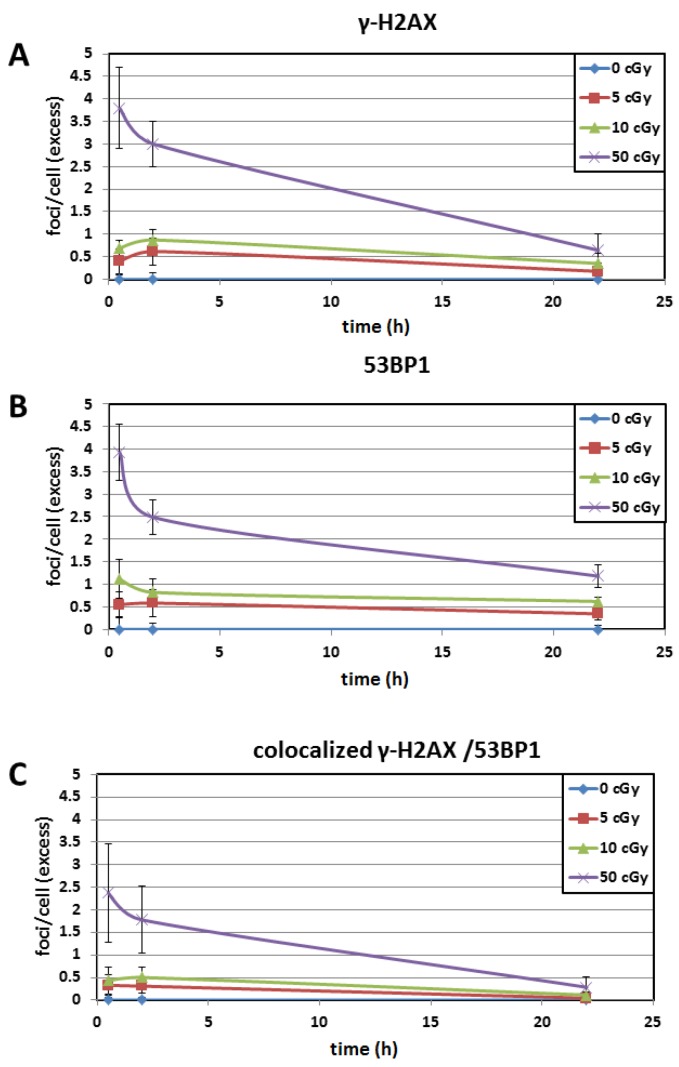
Time kinetics of IRIF. DNA repair foci were measured with: (**A**) γH2AX, (**B**) 53BP1, and (**C**) co-localized γH2AX/53BP1 after irradiation with doses of 0, 5, 10, and 50 cGy. Data show the highest levels at 30 min for: (**A**) γH2AX foci at all doses; (**B**) 53BP1 and (**C**) co-localized γH2AX/53BP1 at 50 cGy. The dose of 50 cGy causes a more rapid decrease in the level of both γH2AX and 53BP1 foci from 30 min to 2 h and then to 22 h as compared to lower doses. This data demonstrates that IRIF induced by low doses (5 and 10 cGy) persist longer than those induced by the higher dose of 50 cGy.

In general, no differences were observed between IRIF at 30 min and 2 h post-irradiation (Table 4, Figure 5A–C). The only exception was statistically significantly decreased in 53BP1 foci induced by 50 cGy (Table 4). Thus, the dependence of IRIF on post-irradiation time as established by multifactorial multivariate ANOVA and described above was stipulated by a significant decrease of IRIF at 22 h. Indeed, further comparison of primary (30 min and 2 h) and residual (22 h) foci showed significant decrease in both γH2AX and 53BP1 from 30 min/2 h to 22 h post-irradiation with 50 cGy (Table 4). While the co-localized γH2AX/53BP1 foci obviously decreased in-between 30 min/2 h–22 h post-irradiation with 50 cGy (Figure 5C) this decrease was not statistically significant (Table 4). Contrary to the effects at 50 cGy, almost no difference was seen between primary and residual foci measured by all end-points after irradiation with low doses ≤ 10 cGy (*p* > 0.05). In line with previously published data for human fibroblasts [26], the obtained results indicated that residual IRIF induced by low doses ≤ 10 cGy in human lymphocytes persist a longer time in comparison with IRIF induced by a higher doses. Further analysis confirmed a longer persistence of low-dose effects. Indeed, higher percentage of γH2AX foci (86.3%) induced by the merged doses ≤ 10 cGy persisted at the location of the lesion as compared to foci induced by a higher dose (36.2%) (Figure 5A). Similarly, low doses up to 10 cGy induced higher percentage of 53BP1 foci persisting as residual foci (82.0%) in comparison with the dose of 50 cGy (49.4%) (Figure 5B). We did not find any statistically significant differences between primary and residual co-localization of both biomarkers even for the dose of 50 cGy (Figure 5C). To conclude, the overall data indicate longer persistence of residual foci at low doses ≤ 10 cGy than at a higher dose.

### 3.4. PFGE

**Figure 6:**
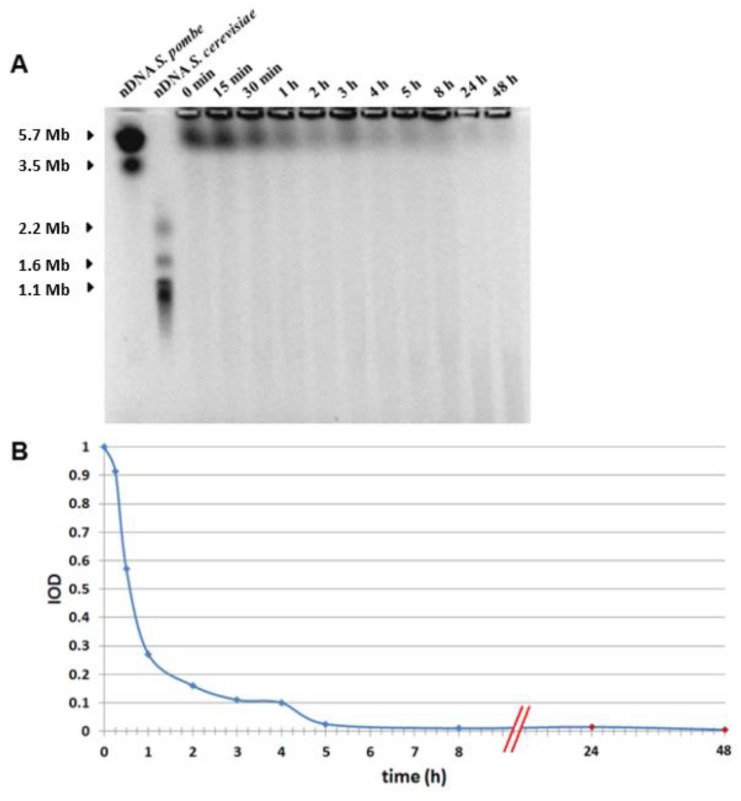
Representative PFGE (A) and qantification of DSB by IOD (B) showing DSB repair. Nuclear DNA was isolated from human lymphocytes at different time points after γ-irradiation with 3 Gy. (**A**) Markers: unirradiated nDNA of S. pombe (line 1) and S. cerevisiae (line 2). Lines 3-13 show the Mb-sized compression zone of fragmented DNA, which decreases with time after irradiation. (**B**) IOD manifests fragmented nuclear DNA due to DSB. Maximum IOD indicating the immediate formation of DSB is observed immediately after irradiation followed by decrease with post-irradiation time due to DSB repair. Almost all DSB are repaired by 5 h post-irradiation.

Kinetics of DSB repair was followed using PFGE to compare with time kinetics of IRIF. The dose of 3 Gy was chosen, which is just slightly above the sensitivity level of the PFGE technique. Contrary to the γH2AX/53BP1 focus formation, the peak value for DSB was observed immediately after irradiation. The kinetics of DSB repair was significantly faster (Figure 6) than kinetics for focus disappearance (Figure 5). Indeed, by 2 h following irradiation with dose of 50 cGy, when ~75% of γH2AX/53BP1 foci persisted in the irradiated cells (Figure 5C), only about ~15% DSB remained as measured by PFGE (Figure 6B). No DSBs were detected at 24 h by PFGE when residual foci constituted approximately 28.71%, 48.31%, and 15.64% as measured with γH2AX, 53BP1, and γH2AX/53BP1, respectively. These data provided further evidence that IRIF kinetics as measured by different molecular markers (γH2AX, 53BP1, and their co-localization) do not completely correlate with DSB repair [30,32,33].

## 4. Discussion

In this study, we focused on analyzing γH2AX and 53BP1 foci, which are generally accepted to be the most sensitive endpoints for enumerating DSB. Post-irradiation time kinetics is an important issue to consider when IRIF are used for biodosimetry. Indeed, primary IRIF usually reach their maximum in-between 15–30 min post-irradiation. Most primary foci disappear with time while some of them, so-called residual foci, persist long time after irradiation when DSB repair has already been completed. While primary foci have been used for biodosimetry, this approach is limited by a very short time window for measurements [9,10,11,18]. On the contrary, residual foci induced by high doses can be used for biodosimetry within a much longer time window, which may last up to 4 weeks post-irradiation [31]. However, no data are available in the literature on dose-response of residual IRIF measured with fluorescent microscopy in human lymphocytes after irradiation with low doses. Here, we measured the level of primary IRIF at the time points of 30 min and 2 h post-irradiation and also residual IRIF at the time point of 22 h. We irradiated cells by low doses 5 cGy and 10 cGy similar to those that humans exposed to during radiological examinations [34,35,36,37]. We also used a moderate dose of 50 cGy for comparison of our measurements with available studies [31,32].

Our data for primary γH2AX foci were in line with other studies where human lymphocytes were irradiated by doses up to: 10 cGy [20]; 20 cGy [38]; 50 cGy [12,39,40,41,42]. Obtained by us levels of primary 53BP1 and co-localized 53BP1/γH2AX foci were also in agreement with previous studies where primary foci were analyzed in human lymphocytes 30 min post-irradiation with doses up to 50 cGy [12,39,41]. To validate our enumeration of residual foci we compared the obtained data with values from the available studies on residual IRIF in human lymphocytes. In these studies, residual foci induced by the moderate doses ≥ 50 cGy and the high doses ≥ 100 cGy were analyzed by either fluorescent microscopy [31,32] or imaging flow cytometry [43]. At the dose of 50 cGy, which has been used in our and available studies, the same level of both γH2AX and 53BP1 residual foci were detected. On the other hand, the level of enumerated residual γH2AX foci obtained by imaging flow cytometry [43] was lower in comparison with our data obtained by fluorescent microscopy. Of note, the same difference was evident between data obtained by imaging flow cytometry [12,43] and fluorescent microscopy (this study) for primary foci. This difference is accounted for the higher resolution of fluorescent microscopy in comparison with imaging flow cytometry [12]. Thus, comparisons of our and literature data have shown that both primary and residual foci were efficiently enumerated in our study.

The dose-response for primary foci fitted well by linear dependence and was in line with previous studies [12,39,40,44]. These data indicated that primary foci enumerated by both molecular markers, 53BP1 and γH2AX, can be used for biodosimetry at low doses given that post-irradiation time is well defined. Importantly, biodosimetry of low doses as based on both direct measurement and extrapolation from high dose effects provides a useful tool for estimating cancer risks [34,35,36,37]. For example, the relative risk for leukemia is 1.0359–1.0595 and lifetime attributable risk for leukemia 21–49/100,000 cases for subjects, which underwent 4D CT and were exposed to 5.4 cGy during this examination [34].

Notably, radiation-induced more 53BP1 foci (2.56 focus/cell/Gy) in comparison with γH2AX (1.40 focus/cell/Gy) or with co-localized γH2AX/53BP1 foci (0.59 focus/cell/Gy) showing that 53BP1 is the more sensitive marker for direct biodosimetry of low doses by residual IRIF. The advantage of this biodosimetry is a prolonged time window, possibly up to 4 weeks [31], within which the measurements may be undertaken.

While dose response for residual IRIF has not previously been studied upon irradiation of human lymphocytes at low doses, there is literature data on response to high doses. Thus, we compared the low-dose-responses obtained here with extrapolations derived by us from those studies, which evaluated dose dependence for γH2AX after high-dose irradiation [31] and 53BP1 [31,32]. This comparison suggested that low-dose effects could be extrapolated from high doses by using γH2AX residual foci. On the other hand, the low-dose dependence of 53BP1 foci enumerated in our study outreached the dose-response obtained by extrapolation from high doses (1.42 ± 0.08 focus/cell/Gy [31]) and 1.15 focus/cell/Gy [32]). This result is accounted for longer persistence of low-dose induced 53BP1 foci as will be discussed below. Such behavior of 53BP1 foci limits biodosimetry of low doses by extrapolation from the dose response obtained at high doses using 53BP1 residual foci.

Although the enumeration of IRIF with γH2AX/53BP1 molecular markers represents a valuable tool for biodosimetry in vitro, the question remains whether this approach could be useful in vivo conditions. A recent study reported data obtained from breast cancer patients, who underwent fractional radiotherapy, 2 Gy per fraction 5 days a week, to a total dose of 50 Gy [7]. Peripheral blood was collected before radiotherapy and 24 h after 1st, 5th 10th day of radiotherapy. The total dose in blood was estimated to be 2 cGy in each fraction. While a statistically significant increase in 53BP1 was found after the first fraction, no further accumulation of residual IRIF was observed till the end of radiotherapy, when accumulated total dose in blood would be about 20 cGy [7]. This finding supported the notion that residual foci represent a marker for cells undergoing apoptosis [32]. Regardless validity of this notion, the data obtained in the in vivo study [7] indicate that, in contrast to the in vitro situation, low-dose induced foci do not reside in irradiated lymphocytes.

It is generally accepted that IRIF at their maximum at 15–30 min post-irradiation is a relevant marker for the enumeration of DSB. In some studies, the kinetics of DNA repair foci is also used for estimation of DSB repair process while the difference in these two kinetics in irradiated human fibroblasts was previously reported [30]. Thus, we aimed to compare the kinetics of DSB repair with kinetics of IRIF in human lymphocytes, which represent one of the most preferential cell types for biodosimetry. Using PFGE we measured DSB during 24 h post-irradiation. PFGE results showed that radiation-induced DSB almost vanished by 5 h post-irradiation. At the same time, 86.3% and 36.2% of γH2AX and 82.0% and 49.4% of 53BP1 foci induced by doses of ≥10 cGy and ≥50 cGy, correspondently, persisted up to 22 h. Since the proportion of remaining residual foci may depend on the dose, we also analyzed literature data for doses comparable with 3 Gy used in our PFGE analysis. In a study by Vasilyev et al. [29] about 18.7% and 90.2% residual γH2AX and 53BP1 foci, correspondently, remained in human lymphocytes 18 h post-irradiation with 2 Gy. The comparison of PFGE data for DSB induced by high dose (3 Gy) with IRIF analysis on a high dose (2 Gy) [29] indicated that residual IRIF persist even after DSB repair has been completed. In combination, the available data strongly support the notion that usage of DNA repair foci as the endpoint for assessment of DSB repair kinetics is significantly limited.

Our data indicated that low-dose induced residual 53BP1 foci may persist longer time than those induced by a higher dose. To further analyze this issue, we compared the level of primary foci (30 min and 2 h post-irradiation) with residual foci (22 h post-irradiation). The level of primary and residual γH2AX and 53BP1 foci induced by low doses (either 5 or 10 cGy) did not differ, while the level of residual foci was significantly lower at the moderate dose of 50 cGy. These results provided evidence that IRIF induced in human lymphocytes by low doses ≤ 10 cGy persist longer on the DSB loci in comparison with IRIF induced by the higher doses. Grudzenski et al. have previously reported strong dependence of IRIF persistence on dose in human fibroblasts where the kinetics of γH2AX and pATM foci loss was substantially compromised after irradiation with doses ≤ 1 cGy [26]. Human cell lines were shown to be several times more sensitive to low doses than expected based on data obtained at higher doses [45]. This phenomenon is most pronounced for cells irradiated in G2 and known as low-dose hyper-radiosensitivity. An inefficient G2/M checkpoint activation could provide an explanation for low-dose hyper-radiosensitivity [46,47,48]. In our study, lymphocytes were irradiated in G0 and the underlying reason for increased radiosensitivity remains to be investigated.

## 5. Conclusions

Primary γH2AX, 53BP1 foci, and their co-localization represent valuable markers for biodosimetry of low doses, but their usefulness is limited by the short time window. Residual γH2AX and 53BP1 foci are the most useful markers for biodosimetry in vitro. Effects of low doses can be extrapolated from a higher doses using γH2AX residual foci while γH2AX/53BP1 foci are valuable markers for evaluation of initial DSB induced by ionizing radiation. Residual IRIF induced by low doses persist longer time than those induced by higher doses.

## Figures and Tables

**Table 1:** Statistical testing dependence of the DNA repair foci and γH2AX pan-staining on dose, post-irradiation time, and treatment with the DNaseI

Variables				Multifactorial ANOVA		Categorical Factors			*p*-Values Adjusted by FDR		
γH2AX *	53BP1 *	γH2AX/53BP1 *	Pan-st. (%)	Multivariate	Univariate	Time	Dose	Treatment	Time	Dose	Treatment
+	+	+	-	+	-	all	+	+	0.00000	0.00000	0.74331
+	-	-	-	-	+	all	+	+	0.00000	0.00000	0.65262
-	+	-	-	-	+	all	+	+	0.00000	0.00000	0.92818
-	-	+	-	-	+	all	+	+	0.00006	0.00000	0.96429
-	-	-	+	-	+	all	+	+	0.10375	0.93183	0.70285
+	+	+	-	+	-	30 min	+	+	-	0.00000	0.90202
+	-	-	-	-	+	30 min	+	+	-	0.00000	1.00000
-	+	-	-	-	+	30 min	+	+	-	0.00000	1.00000
-	-	+	-	-	+	30 min	+	+	-	0.00011	1.00000
-	-	-	+	-	+	30 min	+	+	-	0.84416	1.00000
+	+	+	-	+	-	2 h	+	+	-	0.00000	0.15215
+	-	-	-	-	+	2 h	+	+	-	0.00000	0.19180
-	+	-	-	-	+	2 h	+	+	-	0.00000	0.14320
-	-	+	-	-	+	2 h	+	+	-	0.00006	0.83501
-	-	-	+	-	+	2 h	+	+	-	0.91106	0.70876
+	+	+	-	+	-	22 h	+	+	-	0.00000	1.00000
+	-	-	-	-	+	22 h	+	+	-	0.00000	1.00000
-	+	-	-	-	+	22 h	+	+	-	0.00000	1.00000
-	-	+	-	-	+	22 h	+	+	-	0.00000	0.97244
-	-	-	+	-	+	22 h	+	+	-	0.92655	0.64110

* mean, foci per cell; + the data were included in analysis; - the data were not included in analysis. Statistically significant values are marked red.

**Table 2:** Comparison of DNA repair foci and pan-staining in irradiated and control cells. Effect of irradiation was statistically compared with the sham-irradiated sample at the same time-points after radiation using a *t*-test adjusted for multiple comparisons by FDR. Statistically significant values are marked in red.

FDR		*p*-Values Obtained by *t*-Test Adjusted by FDR			
Time	Dose	γH2AX	53BP1	γH2AX/53BP1	Pan-Staining
0.5 h	5 cGy	0.02	0.02	0.02	0.634
	10 cGy	0.0001	0.002	0.02	0.641
	50 cGy	0.00002	0.000002	0.002	0.572
2 h	5c Gy	0.004	0.006	0.006	0.705
	10 cGy	0.0002	0.001	0.002	0.767
	50 cGy	0.000002	0.000004	0.001	0.635
22 h	5 cGy	0.147	0.003	0.364	0.962
	10 cGy	0.02	0.00001	0.02	0.912
	50 cGy	0.008	0.00001	0.03	0.772

**Table 3:** Slope of linear response (either up to dose of 10 cGy or up to 50 cGy) for γH2AX, 53BP1, and co-localized γH2AX/53BP1 as measured at different post irradiation times.

Dose Response	Time	γH2AX	53BP1	γH2AX/53BP1
up to 10 cGy	**30 min**	y = 0.0712x	y = 0.1114x	y = 0.0476x
	**2 h**	y = 0.0892x	y = 0.0948x	y = 0.0524x
	**22 h**	y = 0.0354x	y = 0.0635x	y = 0.0106x
up to 50 cGy	**30 min**	y = 0.0756x	y = 0.08x	y = 0.0475x
	**2 h**	y = 0.0618x	y = 0.0516x	y = 0.0364x
	**22 h**	y = 0.014x	y = 0.0256x	y = 0.0059x

**Table 4:** Comparison of γH2AX, 53BP1 and co-localized γH2AX/53BP1 foci between post-irradiation time points. Statistically significant values are marked in red.

		*p*-Value Obtained by Fisher’s LSD Test								
		γH2AX			53BP1			Co-Localization		
		30 min	2 h	22 h	30 min	2 h	22 h	30 min	2 h	22 h
0 cGy	30 min	-	0.558	0.484	-	0.366	0.739	-	0.896	0.459
	2 h	0.558	-	0.749	0.366	-	0.113	0.896	-	0.540
	22 h	0.484	0.749	-	0.739	0.113	-	0.459	0.540	-
5 cGy	30 min	-	0.330	0.826	-	0.561	0.471	-	0.950	0.255
	2 h	0.330	-	0.355	0.561	-	0.178	0.950	-	0.146
	22 h	0.826	0.355	-	0.471	0.178	-	0.255	0.146	-
10 cGy	30 min	-	0.138	0.537	-	0.591	0.316	-	0.775	0.251
	2 h	0.138	-	0.599	0.591	-	0.078	0.775	-	0.062
	22 h	0.537	0.599	-	0.316	0.078	-	0.251	0.062	-
50 cGy	30 min	-	0.236	0.008	-	0.039	0.003	-	0.558	0.063
	2 h	0.236	-	0.005	0.039	-	0.002	0.558	-	0.061
	22 h	0.008	0.005	-	0.003	0.002	-	0.063	0.061	-

## References

[1] Bekker-Jensen S., Lukas C., Kitagawa R., Melander F., Kastan M.B., Bartek J., Lukas J. (2006). Spatial organization of the mammalian genome surveillance machinery in response to DNA strand breaks. J. Cell Biol..

[2] Rogakou E.P., Boon C., Redon C., Bonner W.M. (1999). Megabase chromatin domains involved in DNA double-strand breaks in vivo. J. Cell Biol..

[3] Rothkamm K., Lobrich M. (2003). Evidence for a lack of DNA double-strand break repair in human cells exposed to very low x-ray doses. Proc. Natl. Acad. Sci. USA.

[4] Rothkamm K., Balroop S., Shekhdar J., Fernie P., Goh V. (2007). Leukocyte DNA damage after multi-detector row CT: A quantitative biomarker of low-level radiation exposure. Radiology.

[5] Depuydt J., Baert A., Vandersickel V., Thierens H., Vral A. (2013). Relative biological effectiveness of mammography X-rays at the level of DNA and chromosomes in lymphocytes. Int. J. Radiat. Biol..

[6] Vandevoorde C., Gomolka M., Roessler U., Samaga D., Lindholm C., Fernet M., Hall J., Pernot E., El-Saghire H., Baatout S. (2015). EPI-CT: In vitro assessment of the applicability of the γ-H2AX-foci assay as cellular biomarker for exposure in a multicentre study of children in diagnostic radiology. Int. J. Radiat. Biol..

[7] Markova E., Somsedikova A., Vasilyev S., Pobijakova M., Lackova A., Lukacko P., Belyaev I. (2015). DNA repair foci and late apoptosis/necrosis in peripheral blood lymphocytes of breast cancer patients undergoing radiotherapy. Int. J. Radiat. Biol..

[8] Ivashkevich A.N., Martin O.A., Smith A.J., Redon C.E., Bonner W.M., Martin R.F., Lobachevsky P.N. (2011). ΓH2AX foci as a measure of DNA damage: A computational approach to automatic analysis. Mutat. Res..

[9] Barnard S., Ainsbury E., Al-Hafidh J., Hadjidekova V., Hristova R., Lindholm C., Gil O.M., Moquet J., Moreno M., Rößler U. (2014). The first γ-H2AX biodosimetry intercomparison exercise of the developing European biodosimetry network RENEB. Radiat. Prot. Dosim..

[10] Viau M., Testard I., Shim G., Morat L., Normil M.D., Hempel W.M., Sabatier L. (2015). Global quantification of γH2AX as a triage tool for the rapid estimation of received dose in the event of accidental radiation exposure. Mutat. Res. Genet. Toxicol. Environ. Mutagen..

[11] Lamkowski A., Forcheron F., Agay D., Ahmed E.A., Drouet M., Meineke V., Scherthan H. (2014). DNA damage focus analysis in blood samples of minipigs reveals acute partial body irradiation. PLoS ONE.

[12] Durdik M., Kosik P., Gursky J., Vokalova L., Markova E., Belyaev I. (2015). Imaging flow cytometry as a sensitive tool to detect low-dose-induced DNA damage by analyzing 53BP1 and γH2AX foci in human lymphocytes. Cytom. Part A J. Int. Soc. Anal. Cytol..

[13] Horn S., Barnard S., Rothkamm K. (2011). Γ-H2AX-based dose estimation for whole and partial body radiation exposure. PLoS ONE.

[14] Wojewodzka M., Sommer S., Kruszewski M., Sikorska K., Lewicki M., Lisowska H., Wegierek-Ciuk A., Kowalska M., Lankoff A. (2015). Defining Blood Processing Parameters for Optimal Detection of γ-H2AX Foci: A Small Blood Volume Method. Radiat. Res..

[15] Bouwman P., Aly A., Escandell J.M., Pieterse M., Bartkova J., van der Gulden H., Hiddingh S., Thanasoula M., Kulkarni A., Yang Q. (2010). 53BP1 loss rescues BRCA1 deficiency and is associated with triple-negative and BRCA-mutated breast cancers. Nat. Struct. Mol. Biol..

[16] Bunting S.F., Callen E., Wong N., Chen H.T., Polato F., Gunn A., Bothmer A., Feldhahn N., Fernandez-Capetillo O., Cao L. (2010). 53BP1 inhibits homologous recombination in Brca1-deficient cells by blocking resection of DNA breaks. Cell.

[17] Chapman J.R., Sossick A.J., Boulton S.J., Jackson S.P. (2012). BRCA1-associated exclusion of 53BP1 from DNA damage sites underlies temporal control of DNA repair. J. Cell Sci..

[18] Belyaev I.Y. (2010). Radiation-induced DNA repair foci: Spatio-temporal aspects of formation, application for assessment of radiosensitivity and biological dosimetry. Mutat. Res..

[19] Lorat Y., Fleckenstein J., Görlinger P., Rübe C., Rübe C. (2019). Concomitant chemotherapy increases radiotherapy-mediated DNA-damage in peripheral blood lymphocytes. BioRxiv.

[20] Kuefner M.A., Brand M., Engert C., Kappey H., Uder M., Distel L.V. (2013). The effect of calyculin A on the dephosphorylation of the histone γ-H2AX after formation of X-ray-induced DNA double-strand breaks in human blood lymphocytes. Int. J. Radiat. Biol..

[21] Brand M., Sommer M., Achenbach S., Anders K., Lell M., Lobrich M., Uder M., Kuefner M.A. (2012). X-ray induced DNA double-strand breaks in coronary CT angiography: Comparison of sequential, low-pitch helical and high-pitch helical data acquisition. Eur. J. Radiol..

[22] Lobrich M., Rief N., Kuhne M., Heckmann M., Fleckenstein J., Rube C., Uder M. (2005). In vivo formation and repair of DNA double-strand breaks after computed tomography examinations. Proc. Natl. Acad. Sci. USA.

[23] Chauhan V., Wilkins R.C. (2019). A comprehensive review of the literature on the biological effects from dental X-ray exposures. Int. J. Radiat. Biol..

[24] Rehani M.M., Yang K., Melick E.R., Heil J., Salat D., Sensakovic W.F., Liu B. (2019). Patients undergoing recurrent CT scans: Assessing the magnitude. Eur. Radiol..

[25] Janosikova L., Juricekova M., Horvathova M., Nikodemova D., Klepanec A., Salat D. (2019). Risk Evaluation in the Low-Dose Range Ct for Radiation-Exposed Children, Based on DNA Damage. Radiat. Prot. Dosim..

[26] Grudzenski S., Raths A., Conrad S., Rube C.E., Lobrich M. (2010). Inducible response required for repair of low-dose radiation damage in human fibroblasts. Proc. Natl. Acad. Sci. USA.

[27] Ganassi E.E., Zaichkina S.I., Rozanova O.M. (1989). Modeling, using nucleases, of the effect of radiation on Chinese hamster fibroblasts. On the role of single-stranded DNA breaks, induced by DNAase I, in the initiation of chromosome aberrations. Radiobiologiia.

[28] Zhao X., Yang G., Toyooka T., Ibuki Y. (2015). New mechanism of γ-H2AX generation: Surfactant-induced actin disruption causes deoxyribonuclease I translocation to the nucleus and forms DNA double-strand breaks. Mutat. Res. Genetic Toxicol. Environ. Mutagen..

[29] Vasilyev S.A., Kubes M., Markova E., Belyaev I. (2013). DNA damage response in CD133 + stem/progenitor cells from umbilical cord blood: Low level of endogenous foci and high recruitment of 53BP1. Int. J. Radiat. Biol..

[30] Markova E., Schultz N., Belyaev I.Y. (2007). Kinetics and dose-response of residual 53BP1/γ-H2AX foci: Co-localization, relationship with DSB repair and clonogenic survival. Int. J. Radiat. Biol..

[31] Markova E., Torudd J., Belyaev I. (2011). Long time persistence of residual 53BP1/γ-H2AX foci in human lymphocytes in relationship to apoptosis, chromatin condensation and biological dosimetry. Int. J. Radiat. Biol..

[32] Torudd J., Protopopova M., Sarimov R., Nygren J., Eriksson S., Markova E., Chovanec M., Selivanova G., Belyaev I.Y. (2005). Dose-response for radiation-induced apoptosis, residual 53BP1 foci and DNA-loop relaxation in human lymphocytes. Int. J. Radiat. Biol..

[33] Kinner A., Wu W., Staudt C., Iliakis G. (2008). Γ-H2AX in recognition and signaling of DNA double-strand breaks in the context of chromatin. Nucleic Acids Res..

[34] Hoang J.K., Reiman R.E., Nguyen G.B., Januzis N., Chin B.B., Lowry C., Yoshizumi T.T. (2015). Lifetime Attributable Risk of Cancer from Radiation Exposure During Parathyroid Imaging: Comparison of 4D CT and Parathyroid Scintigraphy. AJR Am. J. Roentgenol..

[35] Kim S., Yoshizumi T.T., Frush D.P., Toncheva G., Yin F.F. (2010). Radiation dose from cone beam CT in a pediatric phantom: Risk estimation of cancer incidence. AJR Am. J. Roentgenol..

[36] Bagherzadeh S., Jabbari N., Khalkhali H.R. (2018). Estimation of lifetime attributable risks (LARs) of cancer associated with abdominopelvic radiotherapy treatment planning computed tomography (CT) simulations. Int. J. Radiat. Biol..

[37] Smith-Bindman R., Lipson J., Marcus R., Kim K.P., Mahesh M., Gould R., Berrington de Gonzalez A., Miglioretti D.L. (2009). Radiation dose associated with common computed tomography examinations and the associated lifetime attributable risk of cancer. Arch. Intern. Med..

[38] Vandevoorde C., Vral A., Vandekerckhove B., Philippe J., Thierens H. (2016). Radiation Sensitivity of Human CD34(+) Cells Versus Peripheral Blood T Lymphocytes of Newborns and Adults: DNA Repair and Mutagenic Effects. Radiat. Res..

[39] Jakl L., Lobachevsky P., Vokalova L., Durdik M., Markova E., Belyaev I. (2016). Validation of JCountPro software for efficient assessment of ionizing radiation-induced foci in human lymphocytes. Int. J. Radiat. Biol..

[40] Rube C.E., Fricke A., Widmann T.A., Furst T., Madry H., Pfreundschuh M., Rube C. (2011). Accumulation of DNA damage in hematopoietic stem and progenitor cells during human aging. PLoS ONE.

[41] Sorokina S., Markova E., Gursky J., Dobrovodsky J., Belyaev I. (2013). Relative biological efficiency of protons at low and therapeutic doses in induction of 53BP1/γ-H2AX foci in lymphocytes from umbilical cord blood. Int. J. Radiat. Biol..

[42] Wang J., Yin L., Zhang J., Zhang Y., Zhang X., Ding D., Gao Y., Li Q., Chen H. (2016). The profiles of γ-H2AX along with ATM/DNA-PKcs activation in the lymphocytes and granulocytes of rat and human blood exposed to γ rays. Radiat. Environ. Biophys..

[43] Durdik M., Kosik P., Kruzliakova J., Jakl L., Markova E., Belyaev I. (2017). Hematopoietic stem/progenitor cells are less prone to undergo apoptosis than lymphocytes despite similar DNA damage response. Oncotarget.

[44] Roch-Lefevre S., Mandina T., Voisin P., Gaetan G., Mesa J.E., Valente M., Bonnesoeur P., Garcia O., Voisin P., Roy L. (2010). Quantification of γ-H2AX foci in human lymphocytes: A method for biological dosimetry after ionizing radiation exposure. Radiat. Res..

[45] Joiner M.C., Marples B., Lambin P., Short S.C., Turesson I. (2001). Low-dose hypersensitivity: Current status and possible mechanisms. Int. J. Radiat. Oncol. Biol. Phys..

[46] Lobrich M., Jeggo P.A. (2007). The impact of a negligent G2/M checkpoint on genomic instability and cancer induction. Nat. Rev. Cancer.

[47] Marples B., Wouters B.G., Collis S.J., Chalmers A.J., Joiner M.C. (2004). Low-dose hyper-radiosensitivity: A consequence of ineffective cell cycle arrest of radiation-damaged G2-phase cells. Radiat. Res..

[48] Short S.C., Woodcock M., Marples B., Joiner M.C. (2003). Effects of cell cycle phase on low-dose hyper-radiosensitivity. Int. J. Radiat. Biol..

